# RF-Sputtered ZnO Nano-Coatings on Polyamide Thin-Film Composite Membranes for Tuned Nanofiltration Selectivity

**DOI:** 10.3390/nano16100598

**Published:** 2026-05-13

**Authors:** Catalina Vargas, Daniel A. Palacio, Jesús Ramírez, Eduardo Pérez-Tijerina, Francisco Solís-Pomar, Abel Fundara-Cruz, Rodrigo Bórquez, Andrés F. Jaramillo, Ángelo Oñate, Luis Pino-Soto, Manuel F. Melendrez

**Affiliations:** 1 Departmento de Ingeniería Química, Universidad de Concepción, Edmundo Larenas 219, Box 160-C, Concepción 4070409, Chile; catavargas@udec.cl (C.V.); rborquez@udec.cl (R.B.); 2 Departamento de Polímeros, Facultad de Ciencias Químicas, Universidad de Concepción, Edmundo Larenas 129, Casilla 160-C, Concepción 4070409, Chile; dapalacio@udec.cl; 3 Facultad de Ingeniería, Universidad San Sebastián, Campus Las Tres Pascualas, Lientur 1457, Concepción 4060000, Chile; jesus.ramirez@uss.cl; 4 Centro de Investigación en Ciencias Físico Matemáticas, Facultad de Ciencias Físico Matemáticas, Universidad Autónoma de Nuevo León, Av. Universidad s/n, San Nicolás de Los Garza 66455, Mexico; eduardo.pereztj@uanl.edu.mx (E.P.-T.); francisco.solispm@uanl.edu.mx (F.S.-P.); elpidio.fundora@gmail.com (A.F.-C.); 5 Department of Mechanical Engineering, Universidad de La Frontera, 01145 Francisco Salazar, Temuco 4780000, Chile; andresfelipe.jaramillo@ufrontera.cl; 6 Departamento de Ingeniería Mecánica, Universidad de Córdoba, Cr 6 #76-103, Montería 230002, Colombia; 7 Departamento de Materiales, Universidad de Concepción, 270 Edmundo Larenas, Box 160-C, Concepción 4070409, Chile; aonates@udec.cl; 8 Facultad de Ciencias de la Rehabilitación y de Calidad de Vida, Universidad San Sebastián, Campus Las Tres Pascualas, Lientur 1439, Concepción 4060000, Chile

**Keywords:** nanofiltration, NF90 thin-film composite membrane, ZnO RF sputtering, water contact angle, Spiegler–Kedem model

## Abstract

Water stress is intensifying worldwide, increasing the need for efficient desalination and water purification technologies. Although commercial nanofiltration membranes such as NF90 exhibit high separation performance, their transport properties remain governed by permeability–selectivity trade-offs, and their surface characteristics offer limited tunability for application-specific requirements. Here, a commercial NF90 polyamide thin-film composite nanofiltration membrane was surface modified by depositing ultrathin ZnO coatings via RF sputtering (30–120 s) and evaluated in terms of surface properties, water permeate flux, and NaCl rejection. X-ray diffraction confirmed the formation of crystalline Wurtzite ZnO with preferential (002) orientation. ZnO deposition markedly increased surface hydrophobicity, raising the water contact angle from 52.5 ± 2.0° for the unmodified membrane to 140.4 ± 3.9° after 120 s of deposition. Hydraulic performance decreased after modification, with water permeate flux reduced by approximately 47–50% relative to pristine NF90. In contrast, NaCl rejection increased with ZnO deposition time, particularly at lower operating pressures, and tended to plateau at higher pressures. The Spiegler–Kedem model accurately described experimental rejection-flux behavior. Overall, RF sputtering of ZnO is a feasible post-fabrication route to tune NF membrane selectivity, while introducing a clear trade-off with permeate flux.

## 1. Introduction

Currently, more than one third of the global population lives in water-stressed regions, where freshwater demand exceeds available supply [1]. In this context, membrane-based water treatment technologies have become a promising and increasingly adopted solution. Technological advances and cost reductions have accelerated the deployment of pressure-driven membrane processes, particularly reverse osmosis (RO) and nanofiltration (NF) [2,3]. Most commercially used RO and NF membranes are thin-film composite (TFC) structures composed of three polymeric layers: a polyester nonwoven support, a microporous polysulfone interlayer, and a thin, dense polyamide active layer that governs solute transport and separation [4]. Owing to their high separation efficiency and industrial maturity, these membranes are widely used in desalination and water purification applications.

Despite these advances, a major limitation of membrane-based separations is membrane fouling. Fouling progressively reduces membrane permeability by increasing hydraulic resistance and may promote concentration polarization, which together can impair separation performance and contribute to the partial blockage of flow pathways. This degradation increases operating costs by lowering productivity and requiring more frequent cleaning cycles, ultimately shortening membrane service life [5]. Because membrane modules account for a substantial fraction of total system costs (approximately 20–25%), it has been estimated that around 10% of membranes are replaced annually to maintain optimal operation [6,7].

To tailor membrane performance and interfacial properties, membrane researchers have generally pursued two main strategies. The first is to modify the base polymer formulation and then fabricate membranes using the optimized composition. The second, often simpler and potentially more cost-effective, is to fabricate the membrane from a standard polymer and subsequently modify its surface properties [8]. While the first strategy typically requires application-specific polymer design and process optimization, the second enables the generation of multiple functional variants starting from the same commercial membrane platform [9]. Accordingly, a wide range of post-fabrication modification routes has been investigated for conventional membranes. Among them, the incorporation of metals and metal oxides has received considerable attention due to their potential to improve membrane permeability, selectivity, mechanical robustness, and surface wettability [9]. In many cases, metal-based coatings are applied as thin surface layers to modify membrane interfacial properties and improve stability under operating conditions [10]. In this context, ZnO has emerged as one of the most widely investigated metal oxides for membrane modification because of its ability to alter surface wettability, interfacial properties, and separation behavior. Previous studies have shown that ZnO-based modifications can improve membrane performance under suitable conditions; however, they also reveal important limitations. In incorporation-based routes, membrane performance may depend strongly on nanoparticle loading and dispersion, and excessive ZnO incorporation can adversely affect permeability and overall transport behavior [11]. In addition, depending on the modification route and membrane substrate, coating stability and transport trade-offs remain relevant concerns [12]. Therefore, although ZnO is a promising modifier, a controllable post-fabrication strategy to systematically tune the transport and separation behavior of commercial polyamide nanofiltration membranes remains insufficiently explored [13].

Sputtering is a physical vapor deposition technique in which energetic ions from a plasma bombard a target, ejecting atoms or clusters that subsequently condense on a substrate to form an ultrathin film [14]. In membrane modification, sputtering is attractive as a post-fabrication and solvent-free strategy that enables controlled deposition of thin surface layers without redesigning the bulk membrane structure. Sputtering-based coatings have been explored as a route to modify membrane wettability, surface stability, and separation performance through controlled thin-film deposition. Typically, both the substrate and the target are placed in a high-vacuum chamber where a plasma is generated, enabling the transport of sputtered species from the target to the substrate and, ultimately, film growth [15]. The technique offers good control over coating thickness, surface coverage, phase, and spatial uniformity through deposition parameters such as chamber pressure, gas composition, applied power, target–substrate distance, and deposition time. These features make sputtering particularly suitable for tailoring membrane interfacial properties in a controlled manner. Beyond evaluating desalination-related performance, this study also seeks to clarify how ultrathin ZnO surface deposition modifies the interfacial transport behavior of the membrane. In particular, attention is given to the relationship between ZnO-induced changes in surface properties and the observed trade-off between water permeability and ion rejection.

The novelty of this study lies in the use of RF magnetron sputtering as a controllable post-fabrication strategy to deposit ultrathin ZnO films on a commercial nanofiltration membrane, enabling surface modification without redesigning the bulk membrane structure. Accordingly, this study aims to identify suitable deposition conditions for ZnO film formation and to evaluate how the resulting coatings affect membrane surface characteristics, water permeability, and ion rejection during desalination.

## 2. Materials and Methods

The NF90 membrane (DuPont/FilmTec, Edina, MN, USA) was used as the substrate. NF90 is a nanofiltration thin-film composite (TFC) membrane consisting of a polyamide active layer deposited on a microporous polysulfone intermediate layer with a polyester support. In the present study, the pristine NF90 membrane was used as the reference material for evaluating the effect of ZnO sputter deposition on surface properties, water permeate flux, and NaCl rejection.

### 2.1. RF Sputtering Deposition of ZnO Nano-Coatings

**Figure 1:**
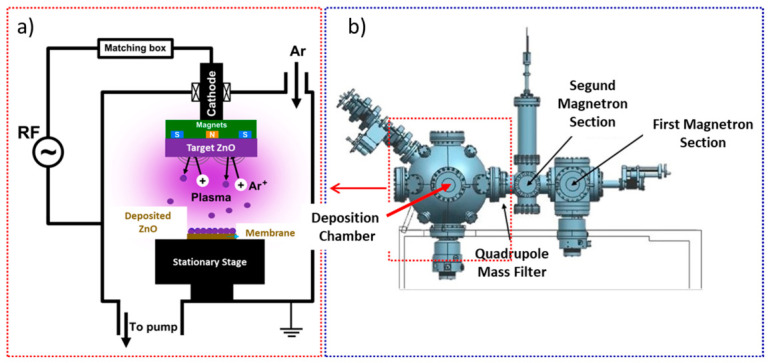
Schematic representation of the ZnO RF magnetron sputtering system and the general architecture of the deposition chamber used in this study. (**a**) Simplified deposition mechanism, showing Ar plasma generation, Ar^+^ ion bombardment of the ZnO target, sputtering of ZnO-related species, and their transport toward the membrane surface placed on a stationary stage. (**b**) General view of the multi-zone vacuum deposition system, including the deposition chamber and the quadrupole mass filter section.

Prior to sputtering, the NF90 membranes were rinsed with deionized water, and their initial water permeability was measured to verify that the pristine membrane performance was within the expected range. The membranes were then dried overnight in an oven at 25 °C before ZnO deposition. After this low-temperature drying step, the membrane coupons were mounted in the sputtering chamber for surface modification. ZnO coatings were deposited on the polyamide surface of NF90 using RF magnetron sputtering in a 10-inch square vacuum chamber (Nanosys 500, Mantis Depositions LTD, Thame, Oxfordshire, UK) (see Figure 1). A ZnO target (99.99% purity) and an RF power source (Advanced Energy, Fort Collins, CO, USA, Cesar RF power generator) were employed, with a target substrate distance of 10 cm. Prior to deposition, the chamber was evacuated to a base pressure of 4 × 10^−5^ Torr. High-purity argon (99.999%) was introduced at 10 sccm, yielding a working pressure of 5.3 × 10^−3^ Torr during deposition. The RF power was set to 100 W, and deposition times of 30, 60, and 120 s were used. All RF sputtering parameters, including RF power, argon flow rate, chamber pressure, and target-to-substrate distance, were kept constant during the experiments. Deposition time was the only parameter systematically varied.

In related sputtering-based nanoparticle deposition architectures reported by Martínez-Carreón and co-workers [16], the deposition system is commonly described as comprising a magnetron sputtering source coupled to an aggregation and transport region and, in some configurations, a quadrupole mass filter positioned between the source and the deposition chamber to enable particle selection prior to deposition (see Figure 1). While the present work employed direct ZnO sputtering onto membrane coupons using a quadrupole-based configuration without cluster mass selection, these reports support the general description of multi-zone vacuum sputtering systems and highlight how gas flow, pressure, and power govern the transport and deposition of sputtered species, as well as the resulting coating formation. During RF magnetron sputtering, argon is introduced into the vacuum chamber and ionized under the applied radio-frequency field, generating a stable plasma. The positively charged Ar^+^ ions are accelerated toward the ZnO target (cathode), producing the ejection of ZnO-related species from the target surface through momentum transfer. The magnetic field generated behind the target confines electrons near the cathode region, thereby increasing the ionization efficiency of the plasma and enhancing the sputtering rate. The sputtered species then travel through the low-pressure gas phase and are transported toward the membrane surface, where they condense and progressively form a ZnO coating. Under the selected deposition conditions, the membrane placed on the stationary stage receives the incoming flux of sputtered species, promoting the formation of a thin surface layer that modifies the membrane topography and surface properties.

### 2.2. Surface and Structural Characterization

Surface morphology was examined by scanning electron microscopy (SEM) using a JEOL JSM-6390LV (JEOL Ltd., Tokyo, Japan). Prior to imaging, samples were sputter-coated with a thin Au layer (~400 Å) to reduce charging effects on the polymeric surface. Representative micrographs were acquired from multiple areas of each membrane to assess surface features before and after ZnO deposition. Crystalline phases were evaluated by X-ray diffraction (XRD) using a PANalytical X’Pert3 Powder diffractometer (Malvern Panalytical, Almelo, The Netherlands). Diffractograms were collected over 2θ = 5–90° and used to identify diffraction peaks associated with crystalline ZnO and the underlying membrane structure. Static water contact angles were measured using a drop shape analyzer (KRÜSS DSA25S; KRÜSS GmbH, Hamburg, Germany) equipped with ADVANCE 1.10 software. A 10 μL droplet of deionized water was gently placed on the membrane surface, and the contact angle was determined by the sessile drop method from the drop profile. Measurements were performed on the membrane active layer side, and images were analyzed using the instrument software to obtain the contact angle values. To relate the apparent contact angle on a rough surface to intrinsic wettability, the Wenzel model was considered [17]:

μ = cos(φ) = rcos(θ)(1)

where θ is the apparent contact angle measured on the rough surface, θ is the intrinsic contact angle on an ideal smooth surface, and *r* is the roughness factor (ratio between the true surface area and the projected area), which can be estimated from AFM data.

Roughness measurements were performed on both pristine and ZnO-modified nanofiltration membranes using an atomic force microscope operated in AC (amplitude-modulation) mode (OmegaScope 1000, AIST-NT Inc., Novato, CA, USA). AFM topography was acquired under non-contact/intermittent contact (tapping) conditions to minimize surface disturbance of the soft polymeric active layer. Images were acquired over a scan area of 10 × 10 μm^2^ on the membrane active layer surface and analyzed using Gwyddion 2.62 software. The roughness parameters reported in this study were calculated from the corresponding topographic height maps obtained for each scanned area, including the root mean square roughness (Rq/RMS), the arithmetic mean roughness (Ra), and the corresponding Wenzel roughness factor (r).

### 2.3. Filtration Tests

Filtration experiments were performed in a dead-end stirred cell (Sterlitech HP4750) from Sterlitech Corporation, Kent, WA, USA. Using an effective membrane area of 15.9 cm^2^. Transmembrane pressure (TMP) was applied using pressurized nitrogen and varied between 2 and 40 bar. All filtration tests were conducted at 24 ± 1 °C. Prior to each experiment, membranes were thoroughly rinsed with distilled water to remove potential residual contaminants from handling and storage. Membranes were then pre-compacted at 10 bar for 1 h to stabilize the membrane structure and minimize flux drift prior to data collection. The water permeate flux density (Jw) was calculated according to Equation (2).

(2) $$J_{W}=\frac{∆V}{A_{m}∆t}$$

where ΔV/Δt is the permeate volume collected per unit time and $A_{m}$ is the effective membrane area. The hydraulic permeability constant $\left(k_{w}\right)$ was obtained from the slope of the water flux pressure relationship and was calculated as:

(3) $$k_{W}=\frac{J_{W}}{∆P}$$

where ΔP is the operational transmembrane pressure difference between the feed and permeate sides. The observed solute rejection ($R_{ob}$) was estimated from conductivity based concentrations measured in the feed and permeate streams according to:

(4) $$R_{ob}\left(\%\right)=\left(1-\frac{C_{ps}}{C_{fs}}\right)\cdot 100$$

In Equation (4), $\left(C_{f}\right)$ denotes the feed concentration (or conductivity based concentration) and ($C_{p}$) denotes the permeate concentration, both determined from conductivity measurements. Because nanofiltration transport can be described using the Spiegler–Kedem (SK) framework, an irreversible thermodynamics-based approach that represents membrane behavior through phenomenological transport coefficients, the SK model was adopted in this study to analyze solute transport in the modified and unmodified membranes. In this model, membrane performance is characterized by two key parameters: the reflection coefficient (*σ*), which quantifies the extent to which the membrane hinders solute convection, and the solute permeability $\left(P_{s}\right)$, which describes diffusive solute transport across the membrane. For a binary system consisting of water and a dissolved solute, where (Jw) and $\left(J_{s}\right)$ are the volumetric water flux and solute flux, respectively, the model can be written as follows [18]:

(5) $$J_{W}=k_{W}\left(∆P-\sigma ∆\pi \right)$$

(6) $$J_{s}=P_{s}∆C_{s}+\left(1-\sigma \right)J_{W}C_{ms}$$

In this equation, (Δπ) represents the osmotic pressure difference across the membrane. (ΔCs) is defined as $C_{m,s}-C_{p,s},$ where ${(C}_{m,s})$ is the solute concentration at the membrane surface and ${(C}_{p,s})$ is the solute concentration in the permeate. According to Equation (6), solute transport comprises the combined contributions of diffusion (driven by the concentration gradient) and convection (associated with solvent flow). The osmotic pressure difference (Δπ) can be estimated using the van’t Hoff equation:

(7) $$∆\pi =\frac{R_{g}T}{m}\left(C_{ms}-C_{s}\right)$$

where ($R_{g}$) is the universal (ideal) gas constant and (T) is the absolute temperature. In the van ’t Hoff relation, the osmotic pressure is commonly expressed in terms of the solute molar concentration; therefore, it is typically calculated using $C\left({mol\cdot m}^{-3}\right)$ and, when needed, an appropriate van ’t Hoff factor for electrolytes. According to the Spiegler–Kedem model, the solute permeability coefficient $\left(P_{s}\right)$ and the reflection coefficient (*σ*) can be obtained by fitting the experimental transport data using the following expressions [14]:

(8) $$R_{R}=\sigma \frac{1-F}{1-\sigma F}$$

(9) $$F=\exp\left(-\frac{1-\sigma }{P_{s}}J_{W}\right)$$

where (F) is a dimensionless parameter (as defined in the model formulation), and ($R_{R})$ denotes the true (actual) solute rejection. The true rejection can also be expressed as follows:

(10) $$R_{R}\left(\%\right)=\left(1-\frac{C_{ps}}{C_{ms}}\right)\cdot 100$$

The model relates the solute concentration at the membrane surface to the solute concentration in the permeate. However, to account for concentration polarization, the transport description must be coupled with film theory, which introduces the mass transfer coefficient ($k_{s}$) for solute back diffusion from the membrane surface to the bulk feed. This coefficient depends on hydrodynamic and system conditions such as feed velocity, temperature, module and cell geometry. Under the film theory framework, the relationship between the solute concentration at the membrane surface and in the permeate can be written as follows [19]:

(11) $$\frac{C_{ms}-C_{ps}}{C_{fs}-C_{ps}}=\exp\left(\frac{J_{v}}{k_{s}}\right)$$

where $\left(k_{s}\right)$ is defined as:

(12) $$k_{s}=\frac{D_{sw}}{\delta }$$

In this equation, ${(D}_{sw})$ is the solute diffusion coefficient in water and (*δ*) is the thickness of the concentration polarization boundary layer. Alternatively, the mass transfer coefficient can be expressed through a Sherwood correlation:

(13) $$k_{s}=\frac{ShD_{sw}}{r_{sc}}$$

where ($r_{sc}$) is the stirred cell radius and ${(S}_{h})$ is the Sherwood number. For the stirred-cell hydrodynamics, ${(S}_{h})$ is given by [20]:

(14) $$Sh=0.285{Re}^{0.55}{Sc}^{0.33}$$

where $\left(R_{e}\right)$ is the Reynolds number and ${(S}_{c})$ is the Schmidt number, defined as:

(15) $$Re=\frac{\rho \omega r_{sc}^{2}}{\mu }$$

(16) $$Sc=\frac{\mu }{\rho D_{sw}}$$

Here, (σ) is the solution density, (μ) is the dynamic viscosity, and (ω) is the angular stirring speed. Finally, combining film theory with the Spiegler–Kedem transport model yields the following expression relating the observed rejection to the transport parameters [21]:

(17) $$\frac{R_{ob}}{1-R_{ob}}=\frac{\sigma }{1-\sigma }\left(1-\exp\left(\frac{{-J}_{v}\left(1-\sigma \right)}{P_{s}}\right)\right)\left(\exp\left(-\frac{J_{v}}{k_{s}}\right)\right)$$

## 3. Results

### 3.1. Physical and Chemical Characterization of ZnO-Modified Membranes

**Figure 2:**
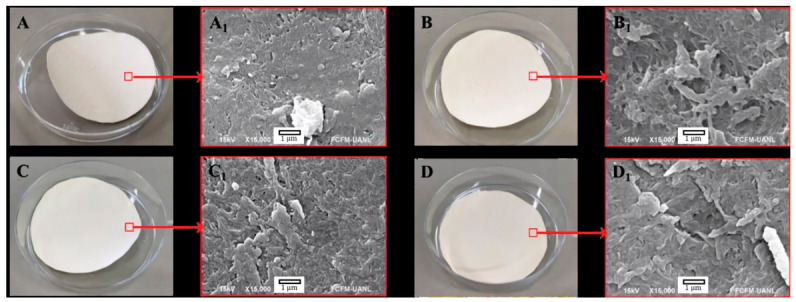
Photographs (**A**–**D**) and SEM surface micrographs of the selected areas (**A1**–**D1**) of polyamide thin-film composite (TFC) nanofiltration membranes before and after ZnO deposition by RF sputtering: (**A**,**A1**) pristine membrane; (**B**,**B1**) ZnO-coated membrane, 30 s; (**C**,**C1**) ZnO-coated membrane, 60 s; and (**D**,**D1**) ZnO-coated membrane, 120 s.

The surface morphology of the membranes is shown in Figure 2. Figure 2A corresponds to the pristine (unmodified) membrane, whereas Figure 2B–D show the membranes after ZnO deposition for 30, 60, and 120 s, respectively. At the magnification employed, the SEM micrographs did not allow direct visualization of a distinct continuous ZnO layer. This is consistent with the ultrathin nature of the sputtered coating and the limited topographic and compositional contrast achievable on the rough polymeric substrate under the selected imaging conditions.

**Figure 3:**
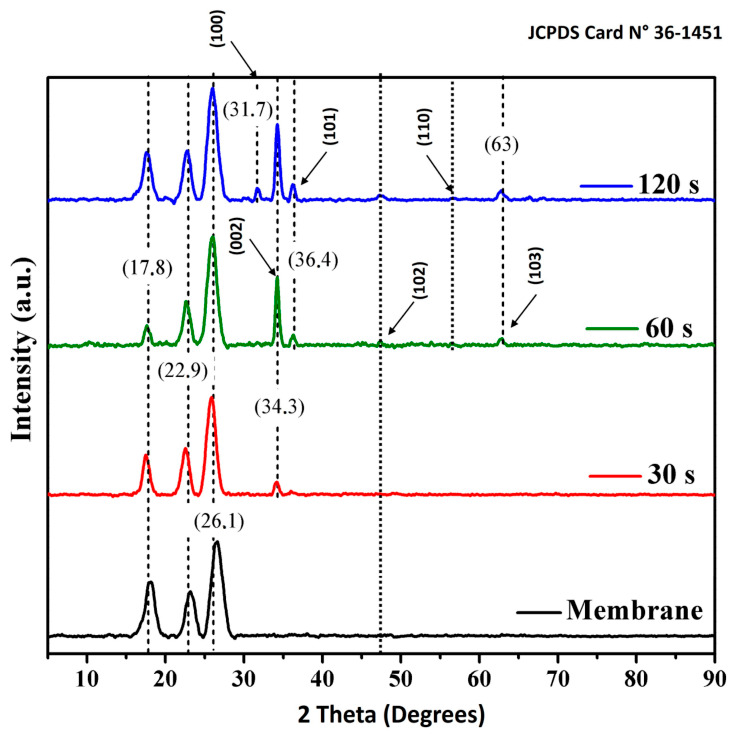
X-ray diffraction (XRD) patterns of pristine and ZnO sputter-modified membranes at different deposition (exposure) times (30, 60, and 120 s).

Surface modification was therefore further examined by X-ray diffraction (XRD) (Figure 3), which provides structural evidence of coating formation. The pristine membrane exhibits diffraction peaks at 2θ = 17.8°, 22.9°, and 26.1°, attributed to crystalline contributions from the polymeric support and assigned to the (O C) reflections for the first two peaks and the (O C N) reflection for the third peak [22]. These features serve as a baseline for identifying additional diffraction signals associated with ZnO after sputtering. These baseline peaks also facilitate comparison with prior reports on Zn-based sputtered coatings on polymeric membranes. For instance, Kacprzyńska-Gołacka et al. [13] reported that magnetron sputtering (MS-PVD) of Zn/ZnO coatings on polyamide membranes leads to measurable changes in surface properties (including wettability and roughness) together with variations in permeate flux, highlighting that even when morphological changes are not prominent at a given SEM magnification, surface and transport properties may still be substantially altered by the deposited layer. In addition, the flux penalty observed in the present work with increasing deposition time is consistent with general trends reported for ZnO-based coatings on porous substrates, where thicker or more developed oxide layers increase hydraulic resistance by partially constricting transport pathways; similar behavior has been documented for aluminum-doped ZnO coatings, where increasing coating thickness produced a systematic decrease in water flux [12]. Therefore, in the present study, ZnO deposition was primarily supported by the XRD results rather than by direct SEM visualization of the coating.

In the modified membranes (Figure 3), four additional diffraction peaks at 2θ = 31.7°, 34.3°, 36.4°, and 63.0° are indexed to the (100), (002), (101), and (103) planes of hexagonal Wurtzite ZnO (PDF#36-1451). A preferential orientation along the (002) plane is observed, consistent with c-axis-oriented ZnO films and commonly attributed to the lower surface energy of this plane [23]. The presence of the (100) and (101) reflections indicates polycrystalline growth, while the appearance of the (103) reflection at deposition times above 60 s suggests enhanced crystallite development and/or increased coating coverage. This preferential (002) texture is consistent with previous reports for ZnO films deposited by RF magnetron sputtering [24].

**Figure 4:**
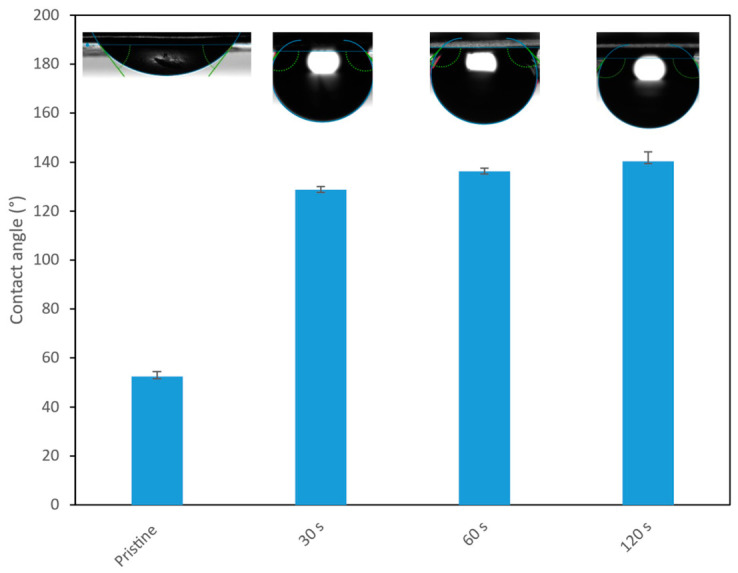
Water contact angle (WCA) images of pristine and ZnO sputter-modified membranes at different deposition times: pristine (unmodified); 30 s; 60 s; 120 s.

Figure 4 summarizes the water contact angle (WCA) measurements of the pristine membrane and the ZnO-modified membranes. The reported values correspond to the average of multiple independent measurements, with error bars indicating the associated dispersion. The pristine polyamide TFC membrane exhibited a WCA of 52.5 ± 2.0°, in agreement with values previously reported for similar membranes [25,26]. After ZnO deposition by RF sputtering, the WCA increased to 128.7 ± 1.3° (30 s), 136.2 ± 1.3° (60 s), and 140.4 ± 3.9° (120 s). This progressive increase with sputtering time suggests gradual modification of the membrane surface, consistent with greater ZnO surface coverage and/or thicker coatings at longer deposition times, resulting in enhanced apparent hydrophobicity, in line with previous reports for sputtered ZnO films and coatings [27]. This wettability change is relevant to transport performance, since a more hydrophobic surface may reduce water affinity at the interface and contribute to lower water uptake. Taken together, the XRD results and the marked increase in water contact angle suggest that RF sputtering mainly modified the outer surface of the membrane.

**Figure 5:**
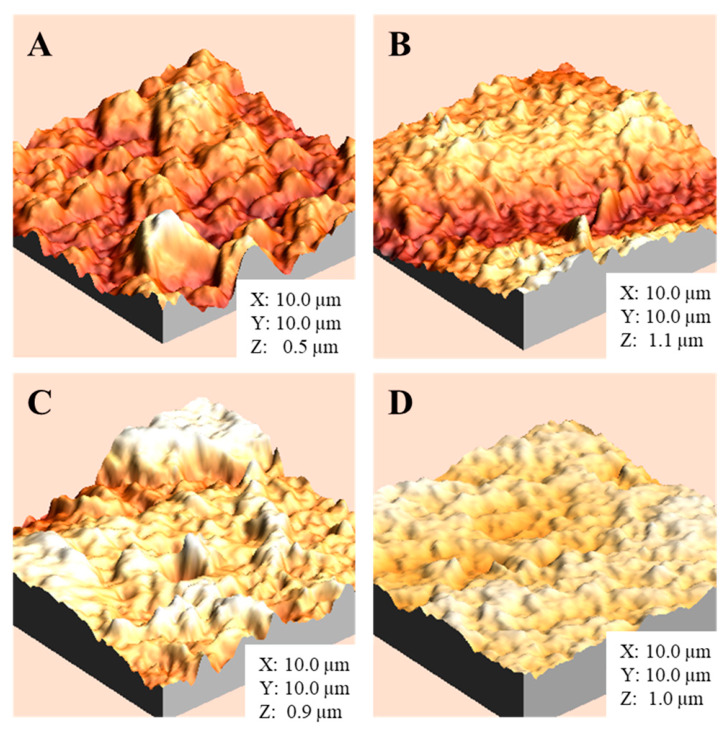
AFM 3D topography images (10 × 10 μm^2^) of (**A**) pristine membrane and ZnO sputter-modified membranes after (**B**) 30 s, (**C**) 60 s, and (**D**) 120 s deposition, showing the evolution of surface texture with sputtering time.

The AFM topography images of the pristine and ZnO sputter-modified membranes are shown in Figure 5, and the quantitative roughness parameters (RMS, Ra) together with the Wenzel roughness factor (r) calculated over a 10 × 10 μm^2^ scan area are summarized in Table 1. The pristine membrane exhibits an RMS roughness of 90.22 nm (Ra = 71.42 nm; r = 1.059). After ZnO sputtering, the surface becomes significantly rougher, with RMS increasing to 230.7 nm at 30 s (Ra = 201.1 nm; r = 1.195) and 249.1 nm at 60 s (Ra = 202.8 nm; r = 1.155), indicating the development of pronounced surface features during the early stages of deposition. Interestingly, at 120 s the roughness decreases (RMS = 174.0 nm; Ra = 144.2 nm; r = 1.096), suggesting a transition from an initially island like growth regime toward coating coalescence and partial filling smoothing of surface valleys as deposition proceeds. This evolution is consistent with the progressive increase in ZnO surface coverage inferred from the XRD results and supports the observed changes in wettability. Overall, the increase in r relative to the pristine membrane is consistent with the higher apparent hydrophobicity observed after ZnO deposition (Figure 4), where surface roughness can amplify intrinsic wetting behavior. The non-monotonic evolution of roughness observed here is consistent with reported growth dynamics of ZnO coatings deposited by RF magnetron sputtering. In particular, Rosa et al. [28], used AFM to track the surface morphology evolution of RF sputtered ZnO and showed that roughness is strongly coupled to the growth stage, evolving from initial fine grained features toward more developed surface structures as deposition proceeds. In addition, Jayatissa et al. [29] reported that processing conditions in RF sputtering can significantly alter ZnO film microstructure and surface roughness, supporting the interpretation that early-stage growth may enhance surface texturing, whereas continued deposition can promote feature coalescence and partial smoothing depending on the evolving film structure. Although AFM confirmed changes in surface topography after sputtering, it does not provide direct evidence of coating thickness or cross-sectional distribution.

### 3.2. Water Permeate Flux Density of Modified Membranes

**Figure 6:**
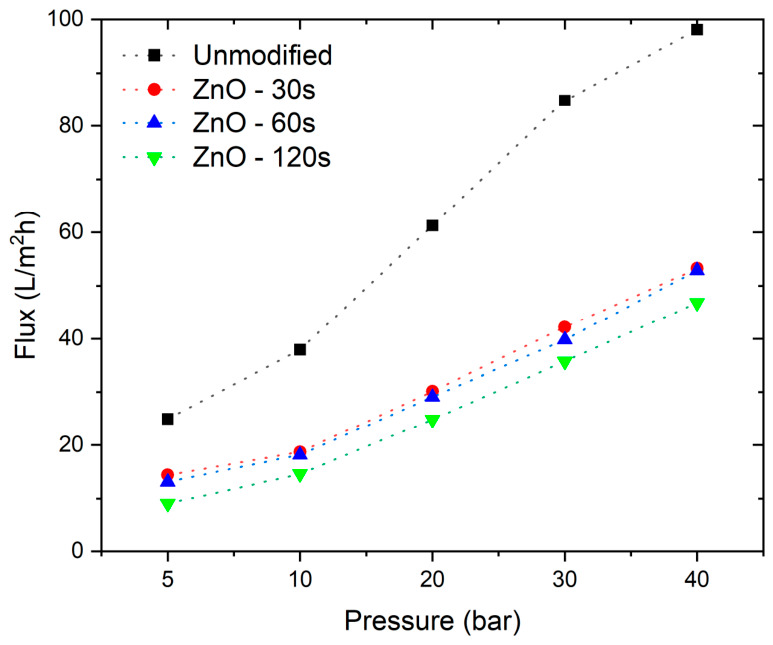
Water permeate flux density as a function of transmembrane pressure for the unmodified commercial NF90 membrane and ZnO sputter-modified membranes prepared with deposition times of 30, 60, and 120 s.

Figure 6 shows the effect of ZnO sputter deposition on the water permeate flux density of the NF90 membrane as a function of transmembrane pressure. Overall, all ZnO-modified membranes exhibit lower flux than the unmodified commercial NF90, indicating that the sputtered coating increases hydraulic resistance to water transport. Among the modified samples, shorter deposition times yield higher fluxes, whereas longer deposition times lead to progressively lower permeate flux density This behavior is consistent with increased ZnO surface coverage at longer sputtering times, which may increase hydraulic resistance and modify the effective transport pathways across the selective layer, thereby decreasing water transport through the membrane. In this regard, the present results differ from many reports in which ZnO was incorporated as dispersed particles or introduced by wet-chemical modification routes, where improvements in hydrophilicity and water permeation are more frequently observed. Similar flux reductions after surface coating have also been reported for metal oxide modified thin-film membranes in related studies [11].

Quantitatively, the permeate flux density decreases by 47.0%, 47.1%, and 49.6% relative to the unmodified NF90 membrane for ZnO deposition times of 30 s, 60 s, and 120 s, respectively. The permeability of the modified membranes is governed by the combined effects of ZnO loading and coverage, wettability, surface texture, and changes in the effective pore structure. In particular, the increase in surface hydrophobicity observed after ZnO deposition (Figure 4) can reduce water surface affinity and contribute to lower flux. Although increased surface roughness may, in principle, increase the effective interfacial area available for transport, the present dataset does not allow the isolated contribution of roughness to be confirmed, because roughness changes occur simultaneously with coating coverage and wettability. Taken together with the XRD evidence of crystalline ZnO formation (Figure 3) and the AFM observed evolution of surface texture (Figure 5 and Table 1), these results indicate that sputtering time is a key control parameter governing the trade-off between surface functionalization and hydraulic performance.

Comparable surface texture changes upon ZnO deposition have been reported for membranes coated using magnetron sputtering. For example, Li et al. [30] deposited ZnO onto polymeric nanofiber membranes by magnetron sputtering and showed that the introduction of a sputtered ZnO layer significantly modifies membrane surface characteristics and filtration behavior, consistent with the idea that early stage deposition can generate pronounced surface features before further deposition consolidates the coating. Similarly, Baig et al. [31] reported ZnO-coated ceramic membranes prepared using sputtering-based deposition, where the ZnO coating alters surface wetting behavior and functional performance, supporting the interpretation that sputtered ZnO layers can measurably modify the interfacial properties that couple surface texture with wettability and transport.

**Figure 7:**
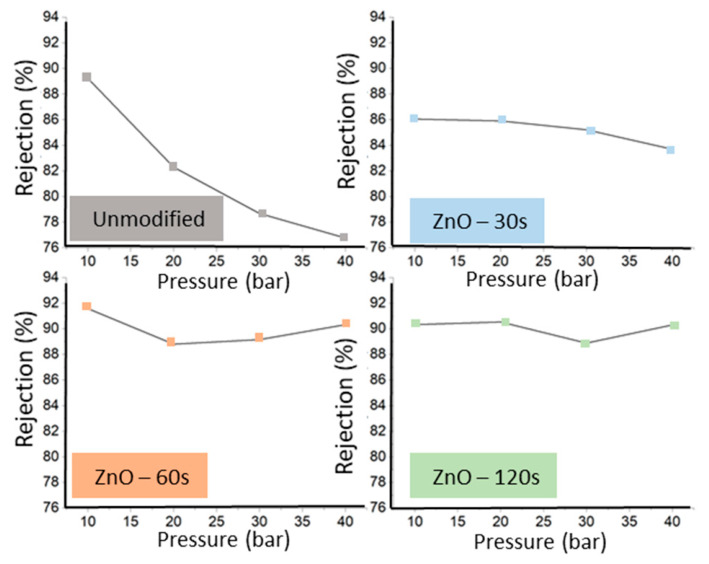
Ion rejection as a function of operating pressure during nanofiltration of a 1 g·L^−1^ NaCl solution using the unmodified commercial NF90 membrane and ZnO sputter-modified membranes prepared with deposition times of 30, 60, and 120 s.

Regarding ion rejection, Figure 7 indicates that ZnO sputtering enhances NaCl rejection relative to the unmodified NF90 membrane, with a clearer improvement as sputtering time increases. This effect is most evident at lower operating pressures (10–20 bar), where longer deposition times consistently yield higher rejection. At higher pressures, the incremental benefit of increasing deposition time becomes less pronounced, and the rejection values for 60 and 120 s converge, suggesting that the selectivity gain approaches a practical plateau under these conditions. This behavior is consistent with the idea that the sputtered ZnO layer progressively modifies the effective transport pathway at the membrane surface, improving solute exclusion up to a point; beyond that, further deposition produces limited additional selectivity while other operating factors dominate the rejection response. Taken together with the concomitant reduction in water permeate flux (Figure 6), the results highlight a time dependent trade-off between increased salt rejection and decreased hydraulic performance. In nanofiltration, solute rejection may be influenced not only by steric effects but also by interfacial properties such as surface charge. Although surface charge was not directly measured in the present work, its potential contribution to the observed rejection behavior should be considered in future studies. Although NaCl rejection increased after ZnO sputtering, the improvement was modest and occurred at the expense of a significant reduction in water permeate flux. Therefore, the present results are better interpreted as a permeability–selectivity trade-off rather than an overall improvement in membrane performance.

Comparable effects of ZnO-based sputtered coatings on membrane performance have been reported in related membrane modification studies. For example, Salih et al. [32] prepared ZnO-coated membranes using sputtering-based routes (including reactive sputtering of ZnO) and showed that introducing a ZnO layer measurably changes membrane surface characteristics and filtration behavior, highlighting that deposition conditions control the balance between functionalization and hydraulic performance. Similarly, Kacprzyńska-Gołacka et al. [13] demonstrated that applying Zn and ZnO coatings onto polyamide membranes leads to clear changes in surface properties (including wettability and topography), underscoring that even thin-sputtered coatings can substantially alter interfacial behavior and, consequently, transport trends.

**Figure 8:**
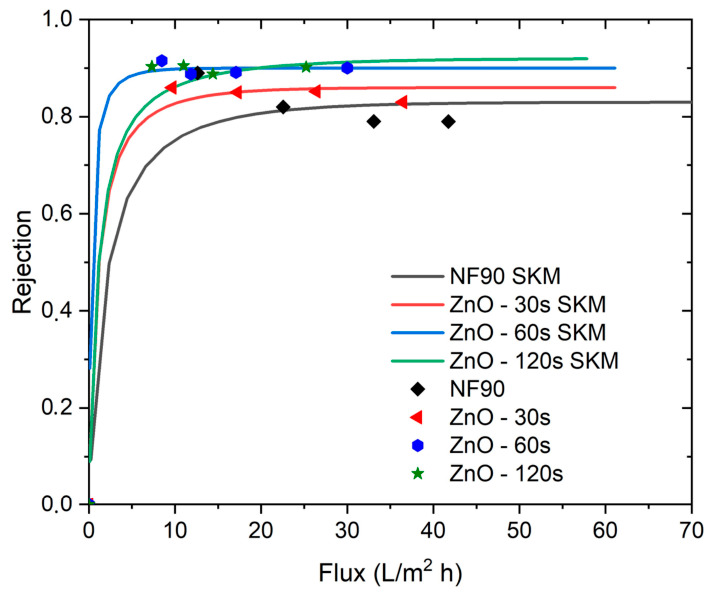
Solute rejection as a function of permeate flux: experimental data and Spiegler–Kedem model (SKM) predictions for the unmodified commercial NF90 membrane and ZnO sputter-modified membranes prepared with deposition times of 30, 60, and 120 s.

The application of the Spiegler–Kedem model to the unmodified NF90 and the ZnO modified membranes is shown in Figure 8, and the fitted transport parameters are summarized in Table 2. The model captures the experimental rejection flux relationship with good agreement over the evaluated range, supporting its suitability for describing NaCl transport in these membranes under the tested conditions. Notably, the fitted reflection coefficient increases systematically from 0.83 (NF90) to 0.86, 0.90, and 0.92 for ZnO deposition times of 30, 60, and 120 s, respectively (Table 2). This monotonic increase is consistent with the experimentally observed enhancement in rejection after ZnO sputtering (Figure 7), indicating that the modified membranes behave as more selective barriers to solute convection as deposition time increases.

In parallel, the fitted solute permeability ($P_{S}$) decreases upon ZnO modification relative to NF90, with the lowest value obtained for the 60 s sample (Table 2). This reduction in $\left(P_{S}\right)$ indicates a lower propensity for solute passage via diffusive transport, which is consistent with the higher rejection levels predicted by the model curves in Figure 8. Together, the increase in (σ) and the reduction in ${(P}_{S})$ support the interpretation that sputtering time progressively shifts the transport balance toward higher solute exclusion, in line with the experimentally observed flux rejection trade-off.

The mass transfer coefficient (k) remains constant across the fitted conditions (Table 2), suggesting that the external mass transfer contribution (concentration polarization term in the coupled model) was effectively similar among the tests, and that the differences in performance are primarily captured by changes in the intrinsic membrane transport parameters (σ) and ${(P}_{S})$. Overall, the combined experimental trends (Figure 6 and Figure 7) and the SK fits (Figure 8 and Table 2) indicate that ZnO sputtering provides a controllable route to tune nanofiltration selectivity, with deposition time acting as a key parameter governing the balance between solute rejection and hydraulic transport. The suitability of the Spiegler–Kedem framework observed in Figure 8 is consistent with prior studies that have successfully applied this irreversible thermodynamics based model to describe nanofiltration performance of commercial membranes under electrolyte separation conditions. For example, Al-Zoubi and Rieger [33] used the Spiegler–Kedem approach for modeling nanofiltration membranes, including commercial NF grades such as NF90, showing that the model parameters can capture the rejection response over a range of operating conditions and provide a compact description of membrane selectivity. Similarly, Mikulášek and Kušnierik [34] reported Spiegler–Kedem-based characterization of nanofiltration membranes and demonstrated that the model can reproduce experimental rejection trends for NaCl type systems when coupled with appropriate mass transfer considerations, supporting the interpretation of rejection flux behavior through the combined contributions of diffusive and convective transport captured by (σ) and ${(P}_{S})$.

## 4. Conclusions

This study evaluated the effect of RF magnetron sputtering of ultrathin ZnO coatings on a commercial NF90 polyamide thin-film composite nanofiltration membrane, focusing on surface and structural changes as well as on desalination-related performance indicators, namely water permeate flux and NaCl rejection. The results showed that RF sputtering enabled the deposition of crystalline ZnO on the NF90 surface without evidence of macroscopic surface damage in the SEM observations, demonstrating the feasibility of this post-fabrication modification route for commercial TFC nanofiltration membranes. XRD confirmed the formation of hexagonal Wurtzite ZnO on the modified membranes, with a preferential (002) orientation and additional reflections assigned to the (100), (101), and (103) planes, the latter becoming detectable at longer deposition times. These findings indicate that sputtering time governs the structural development of the deposited ZnO layer and can therefore be used as a controllable parameter in membrane surface engineering.

ZnO deposition also produced marked changes in membrane interfacial properties. The water contact angle increased from 52.5 ± 2.0° for the unmodified membrane to 128.7 ± 1.3°, 136.2 ± 1.3°, and 140.4 ± 3.9° for 30, 60, and 120 s, respectively, indicating a strong increase in apparent hydrophobicity. AFM results revealed a concurrent evolution in surface roughness, with RMS values increasing at 30–60 s and decreasing at 120 s, consistent with changes in surface texture as deposition proceeded. Together, these results show that RF sputtering can significantly alter the surface characteristics of NF90 membranes in a time-dependent manner. Future work should include direct thickness characterization and spatial uniformity analysis of the sputtered ZnO layer.

In terms of separation performance, all ZnO-modified membranes exhibited lower water permeate flux than pristine NF90, with decreases of 47.0%, 47.1%, and 49.6% for 30, 60, and 120 s, respectively. In contrast, NaCl rejection increased with deposition time, particularly at 10–20 bar, while the improvement tended to plateau when deposition time increased from 60 to 120 s at higher pressures. These results demonstrate a clear trade-off between hydraulic permeability and salt rejection, indicating that the observed increase in NaCl rejection was achieved at the expense of water permeate flux rather than as an overall improvement in membrane performance. From a broader perspective, this finding is relevant because it shows that the separation response of a commercial nanofiltration membrane can be tuned through post-fabrication surface modification without altering the bulk membrane architecture.

The Spiegler–Kedem model provided a good description of the experimental rejection–flux relationship for both the unmodified and ZnO-modified membranes, supporting its applicability for representing solute transport in this system through combined diffusive and convective contributions, as captured by the fitted model parameters. This result reinforces the interpretation that ZnO sputtering affects not only membrane surface properties but also the effective transport response of the membrane. Overall, the present work demonstrates that RF-sputtered ZnO is a feasible and controllable post-fabrication strategy for tuning the surface and transport behavior of NF90 nanofiltration membranes. Although the present study provides a proof-of-concept demonstration, further work is required to assess the practical applicability of this modification under realistic operating conditions. In particular, future studies should include systematic anti-fouling experiments and long-term cross-flow stability tests in order to evaluate the operational durability and application potential of the modified membranes. Future work should also incorporate complementary characterization of surface charge, effective pore structure, and membrane chemical state in order to better correlate surface modification with nanofiltration performance.

## Figures and Tables

**Table 1:** AFM roughness parameters of pristine and ZnO sputter-modified membranes.

Membrane	Scan Area (μm^2^)	Rq (RMS) (nm)	Ra (nm)	Wenzel Factor, r (-)
Unmodified	10 × 10	90.22	71.42	1.059
ZnO-coated (30 s)	10 × 10	230.7	201.1	1.195
ZnO-coated (60 s)	10 × 10	249.1	202.8	1.155
ZnO-coated (120 s)	10 × 10	174.0	144.2	1.096

**Table 2:** Spiegler–Kedem model parameters obtained for the unmodified NF90 membrane and ZnO sputter-modified membranes (30, 60, and 120 s): reflection coefficient σ (-), solute permeability $P_{s}({m\cdot s}^{-1})$, and mass transfer coefficient $k({m\cdot s}^{-1})$.

Membrane	σ (-)	P (m·s^−1^)	k (m·s^−1^)
NF90	0.83	4.86 × 10^−7^	0.011
ZnO 30 s	0.86	2.58 × 10^−7^	0.011
ZnO 60 s	0.90	7.25 × 10^−8^	0.011
ZnO 120 s	0.92	2.85 × 10^−7^	0.011

## Data Availability

The authors confirm that the data supporting the findings of this study are available within the article. In addition, the datasets used and/or analyzed during the current study are available from the corresponding author on reasonable request.
